# Does the risk-taking behaviour of a group influence individual risk-taking behaviour?

**DOI:** 10.1098/rsos.250200

**Published:** 2025-07-16

**Authors:** Asieh Daneshi, Marcel Brass

**Affiliations:** ^1^ Berlin School of Mind and Brain, Department of Psychology, Humboldt University of Berlin, Berlin, Germany; ^2^ Science of Intelligence, Research Cluster of Excellence, Berlin, Germany

**Keywords:** risk-taking behaviour, social contagion, decision-making in the group, conformity

## Abstract

People often take more risks when in groups than when alone, but studying group-based risk-taking can be logistically challenging. This study used an online simulation of the balloon analogue risk task featuring virtual agents to examine how the presence of others influences individual decision-making. Ninety-nine participants completed 160 trials of a balloon inflation task while situated in a virtual room with five computer-generated agents. On each trial, a varying number of agents also inflated balloons and displayed either risk-taking or risk-avoiding behaviour. Results showed that participants’ risk-taking generally increased as more agents co-inflated, and this effect was stronger when the agents behaved riskily, consistent with the risky shift phenomenon. These findings demonstrate the utility of virtual simulations for exploring social influences on risk-related decisions.

## 1. Introduction

Every day, we are confronted with numerous decisions starting from the moment we wake up. The first decision is whether to indulge in a few more minutes of sleep or get up immediately. Sleeping longer seems appealing, but we may end up running late for work. Another decision that almost everybody has to make every day is about passing streets when the traffic light signals red. Even though we know how dangerous it can be, most of us have sometimes contemplated passing the street when the traffic light is red, particularly when we are in a hurry.

Risk-taking can be defined as engaging in actions with the potential for unfavourable outcomes [1]. Given that a lot of our decisions in real life carry a potential for negative consequences and often one cannot ascertain the quality or the probability of these potential negative consequences, taking risks when making decisions is inevitable [2–5].

People assess risks differently based on their unique experiences, values and perceptions [6,7]. Social contexts, personality traits and resistance to peer influence significantly impact risky decisions, with men typically making riskier choices than women [8]. Furthermore, cognitive biases and heuristics also shape how people perceive and respond to risks, often viewing them subjectively based on personal values and experiences [9].

It is well known that decisions are not only shaped by the properties of the decision maker, but also influenced by their social surroundings [10–15]. Many crucial decisions, especially those involving risk, are made within social groups rather than in isolation [15–17]. Research on social conformity has revealed that individuals tend to align their attitudes, beliefs and behaviours with the group [18–24]. When it comes to risky decision-making, individuals likely also rely on the decisions of others because this can be a strategy to deal with risk [25–33,]. Accordingly, it has been proposed that individuals prefer to make risky decisions in the group because in this way the potential adverse outcomes resulting from wrong choices will be distributed among multiple individuals (e.g. when jaywalking, the likelihood of being run over by a car is reduced when walking in a large group). This has been investigated widely under the concepts of responsibility diffusion and interpersonal influence [34–41]. Furthermore, some researchers argued that aligning with the group serves the purpose of gaining additional information that might reduce perceived risk (e.g. if everybody crosses the street safely when the traffic light is red, it cannot be so risky) [42–48]. It is worth noting that there is a third alternative that has received less attention. Many people are more willing to make risky decisions in the presence of others, even if the presence of others does not provide them with any additional information and the consequences of wrong decisions are only directed at them [32,49,50]. More specifically, it has been shown that individuals make riskier decisions in peer groups than alone [44]. It has been shown that people in the mere physical presence of others make riskier decisions, with feelings of security being the psychological mechanism behind this effect [50]. We often feel more confident and emboldened in the presence of others, even when those individuals do not provide any additional information or support [21,51,52]. This phenomenon can be explained by the concept of social presence, which refers to the degree to which an individual feels the presence of others and the associated psychological and behavioural effects [53–56]. When individuals feel a heightened sense of social presence, they may experience a greater sense of mutual understanding and behavioural engagement with others. This can lead to increased confidence in one’s own abilities and a willingness to take on more risk or challenge [54,56].

It is important to acknowledge, however, that the influence of social presence can depend on the specific context and conditions of the decision-making environment. In some situations, the mere physical availability of others may not provide the psychological sense of presence typically associated with this phenomenon. This nuance highlights the need to carefully differentiate between physical presence and the subjective experience of social presence when interpreting its role in risky decision-making.

Real-life examples of risky decision-making have been documented in studies of pedestrian decision-making, such as individuals crossing the street against a red traffic signal [57–62]. Research has shown that people are more likely to engage in this risky behaviour when accompanied by others, particularly if those others also initiate crossing [57,63–66]. The presence of peers can encourage conformity and risk-taking, even in situations where such actions may lead to adverse consequences [18,21,67]. However, investigating the influence of a group on individual risk-taking under controlled experimental conditions is difficult because this requires involving a group of confederates. Therefore, we implemented a virtual group approach to investigate individual risk-taking in a group of virtual agents. Previous research has demonstrated that the presence of others can shape our perceptions, motivations and experiences, even in virtual or online environments [54].

We sought to explore risk-taking behaviour in groups in a dynamic context, where both the agents and participants are engaged in a real-time task within a constantly changing environment. As there are relatively few studies on risk-taking within group settings, we opted to develop one of the experiments originally designed for studying individual risk-taking to explore risk-taking behaviour within a group context. Many researchers have studied how risk-taking behaviours develop by analysing real-life instances of risk-taking [68–76], while others have taken a different approach by studying how people perform in controlled laboratory experiments that simulate risk-taking scenarios [77–82]. The advantage of the laboratory approach is that it simulates risky decisions in everyday life while controlling other factors and assessing only the impact of specific variables of interest. A relatively widely used experimental paradigm to investigate risk-taking under controlled experimental conditions is the ‘balloon analogue risk task’ (BART) introduced by Lejuez *et al*. [83]. In this task, participants have to decide how far to inflate a balloon. Taking risks is initially rewarded up to a certain point (i.e. participants receive points), but after passing that point, taking more risks leads to less favourable results, and participants lose the points they earned in the trial [83]. The results of the BART showed a significant correlation with scores on self-report measures of risk engagement in real-life risky behaviours [83]. A main advantage of the BART is that it allows the investigation of the dynamics of risky decision-making. We developed a group version of the BART to investigate how risk-taking behaviour is influenced by the group when the individual decision and the group decision are independent. The balloon-inflating task has certain characteristics that made it a suitable choice for our study. Firstly, it represents a risky decision-making scenario in a real-world context that can be easily simulated in the laboratory. Secondly, it is repeatable, allowing for the implementation of a large number of trials in a short time interval (a session of the experiment). Additionally, experimental parameters (such as the risk aversion of agents) are quantifiable and can be easily manipulated.

Despite the wealth of research on social influence and risk-taking, few studies have examined how individuals make dynamic, experience-based decisions in the presence of others, especially in settings where group members act independently but concurrently. Most existing studies rely on either static scenarios (e.g. vignettes or questionnaires) or require complex group coordination in laboratory-based setups. These approaches often limit ecological validity or are difficult to scale. Moreover, the role of group behaviour—specifically, how the number of peers and their risk-taking tendencies influence individual behaviour in real time—has not been systematically isolated. The current study addresses this gap by simulating group presence using virtual agents in a dynamic version of the BART, enabling precise manipulation of group characteristics while maintaining individual decision independence.

Here, we investigate the effect of the risk-taking behaviour of the group on individuals’ risk-taking when they make independent decisions in the group. Another factor that we investigated in this study was the number of individuals who are engaged in the same task alongside the participant.

## 2. Material and methods

### 2.1. Participants

Ninety-nine participants (49 female, 50 male, age 29 ± 5.6 years (mean ± s.d.), minimum age 20 and maximum age 40) were recruited via Prolific (https://www.prolific.com/) to take part in this experiment. The experiment protocol was approved by the Humboldt University of Berlin ethical committee board (reference number: 2022-55). Participants were naive with respect to the purpose of the experiment, gave written informed consent to their participation in the experiment and were paid for their participation.

Two participants (both male) were excluded from the analysis because the total time they spent on the experiment was indicated as outliers according to the MAD criterion. We used the effect and sample sizes from a pilot run of the experiment with 20 participants as guidance for an appropriate sample size for this study. An *a priori* power analysis was conducted using G*Power [84] to determine the required sample size for detecting a small effect size in a repeated-measures design. With an effect size of *f* = 0.10, *α* = 0.05, power = 0.85, eight measurements and a nonsphericity correction *ε* = 1, the required sample size was estimated to be *n* = 102. Our final sample of 97 participants exceeded this threshold, indicating adequate power to detect small effects. Data collection was completed prior to analysing the data.

### 2.2. Apparatus and experimental procedure

To take part in this experiment, participants needed to use a computer. Stimuli were generated with Unity (v. 2021.3.11 .f1).

The experimental setup and procedure, number of participants and the data analysis procedure were pre-registered (https://aspredicted.org/CTZ_816).

### 2.3. Experiment design

**Figure 1.**
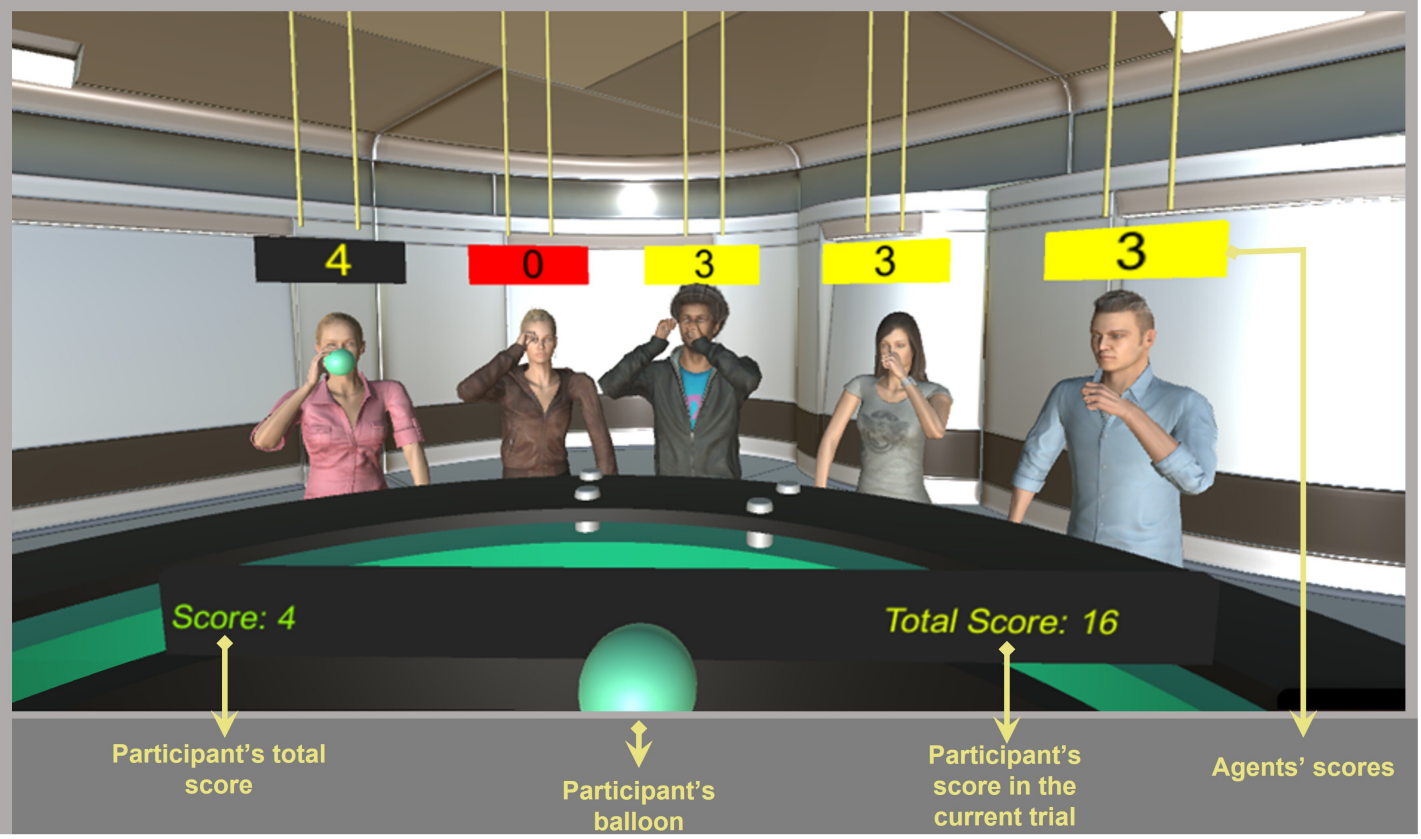
The environment of the experiment. The three agents on the right have quit the ongoing trial and are lowering their hands. Their balloons are deflated, the screens above their heads turned yellow and their score in the ongoing trial is frozen. The balloon of the second female agent from the left has burst, and she is also lowering her hands. The screen above her head turned red and her score reset to zero. The first female agent from the left is still inflating her balloon. (In this trial, the three agents on the right quit almost at the same time. That is why their score is frozen at the same value. However, it is worth noting that this is just a coincidence.)

The aim of our study was to investigate the effect of two factors: the number of agents involved in each trial (agents who inflated their balloons) and the risk-taking behaviour of the agents (high versus low). Participants were instructed to inflate virtual balloons in the presence of five virtual agents (figure 1). No explicit information was provided regarding whether these agents were real humans or computer-generated. Visual realism and human-like movements were used to enhance credibility. To inflate their balloons, they were required to hold down the space key. Once they released the key, the trial ended, and a fixation cross appeared before they initiated the next trial. In summary, each trial started with the participants pressing the space key and ended with them releasing the key or when their balloon burst. No opportunity was given to pause the experiment and resume it again. In 25% of the trials, only the participant inflated his/her balloon. In 25% of the trials, one, in 25% three and in 25% all five agents inflated their balloons alongside the participant. Trials were organized in a pseudo-random order, ensuring that consecutive trials did not involve the same number of agents inflating their balloons. In half of the trials, agents stopped inflating their balloons quite fast (risk-avoiding condition), while in the other half, agents kept inflating their balloons for a longer time (risk-taking condition). The order of risk-taking and risk-avoiding trials was completely random.

**Figure 2.**
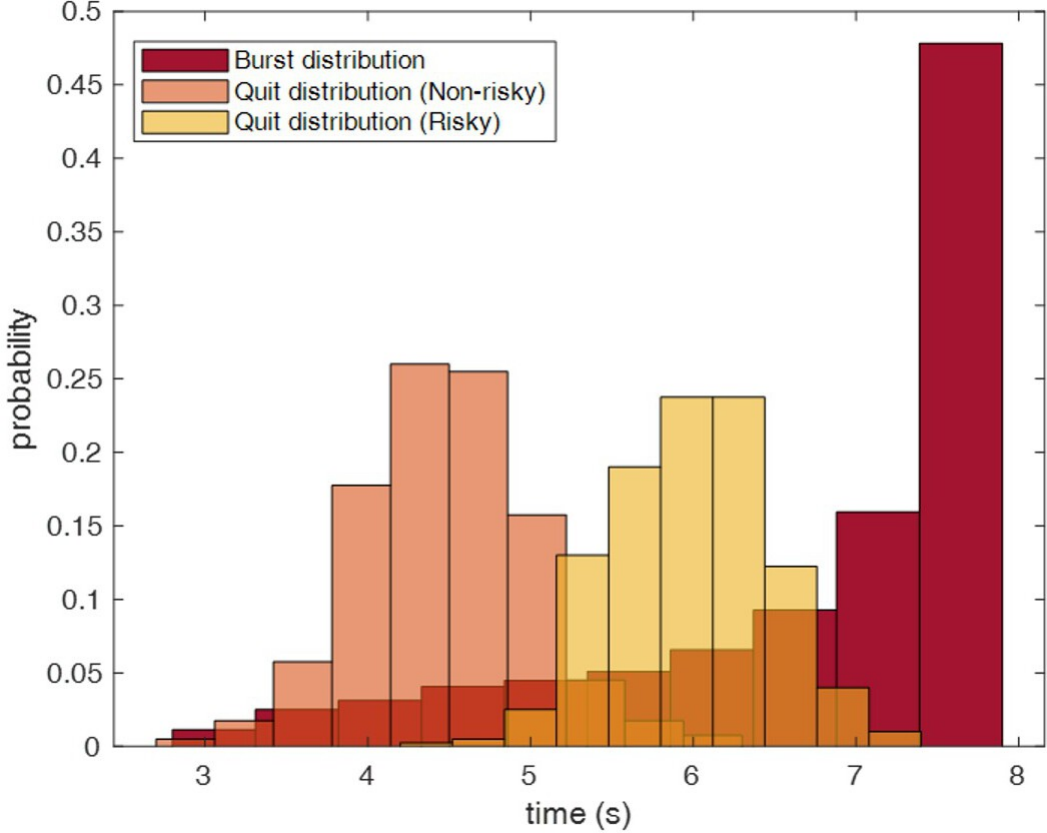
An example of the distribution for burst times (dark red distribution) and quit time for risk-taking (yellow distribution) and risk-avoiding (orange distribution) conditions. The risk-taking condition refers to the trials in which agents kept blowing in their balloons for a longer time, while the risk-avoiding condition refers to the trials in which agents quit early.


The burst time for the participant’s balloon and agents’ balloons was drawn from the distribution in equation (2.1) (e.g. the dark-red distribution in figure 2), and the quit times for risk-taking and risk-avoiding behaviour of the agents were drawn from a Gaussian distribution (equation (2.2)) respectively with mean (μ) equal to 4.5 and equal to 6, and standard deviation (σ) of 0.5 (e.g. respectively, the yellow and the orange distributions in figure 2).

(2.1) $$f\left(t\right)=1-2.25^{-5t},$$

(2.2) $$f(t)=\frac{1}{\sigma *\sqrt{\left(2*\pi \right)}}*e^{-\frac{1}{2}{\left(\frac{t-\mu }{\sigma }\right)}^{2}},$$

where *t* indicates time, and $f\left(t\right)$ is the probability distribution (group risk-taking: high versus low).

A small screen above the head of each agent displayed that agent’s score for each trial (as shown in figure 1). Additionally, in front of the participant, there was a larger screen presenting the participant’s trial score on the left and the total score they had accumulated since the beginning of the experiment on the right. The score in each trial was calculated with the following formula:


(2.3) $$\text{score}=3*\;\text{round}\left(\frac{1.8^{0.5*t}}{2}\right),$$

where *t* is the time elapsed from the beginning of each trial. Since the inflating speed for all the agents and the participant was the same, their scores at each moment were identical unless one of the agents quit or their balloon burst. The participant’s score in each trial was directly tied to the size of their inflated balloon.

Based on the probability distributions for balloon burst times (equation (2.1)) and agents’ quit times (equation (2.2)), we calculated that the optimal time to stop inflating the balloon is after the median of the burst distribution, which corresponds to approximately 6 s after the trial onset.

When one of the agents quit a trial, the screen above his/her head turned yellow and that agent’s score was frozen until the end of that trial. Also, the agent stopped inflating his/her balloon and lowered his/her hands, resulting in the agent’s balloon deflating. If an agent’s balloon burst, a balloon burst sound was triggered and the screen above his/her head turned red and that agent’s score was immediately reset to zero. Additionally, that agent’s balloon immediately disappeared. The rules were the same for the participant, except that in the case the participant quit a trial, the trial was automatically ended after 200 ms.

The participants were informed that in each trial, the quality of the agents’ balloons was the same as the quality of their own balloons. However, in order to avoid the carryover effect, participants were informed that in each trial, participants and involved agents receive new balloons. They were also informed that they would be scored in each trial based on the size of their balloon; the bigger their balloons, the higher their score. However, if their balloon burst, they would lose the score for that ongoing trial. Furthermore, they were instructed that they could earn an additional two euros if they were among the top 10% of all participants.

The experiment consisted of two parts: a practice session and a main session. The practice session was designed to familiarize the participants with the environment of the experiment and their task before they start the main session.

### 2.4. Procedure

#### 2.4.1. Practice session

The practice session of the experiment consisted of 20 trials. In all trials, all five agents were present. In the first four trials, none of the agents inflated their balloons in order to give participants the opportunity to become acquainted with the experiment’s environment without overwhelming them with too much information. In four of the remaining trials, again, none of the agents inflated their balloons; in four trials, only one agent (the middle one in figure 1); in four trials, the three middle agents; and in four trials, all five agents inflated their balloons.

#### 2.4.2. Main session

The main session of the experiment consisted of 160 trials. The structure of the main session of the experiment was exactly like the structure of the last 16 trials in the practice session of the experiment, except that the number of trials in each condition was multiplied by 10. As outlined above, the factors of the number of involved agents (0, 1, 3, 5) and risk-taking behaviour (low and high) were crossed. However, since in the zero agent condition the distinction between low and high risk-taking conditions does not make sense, the condition was neglected in all analyses that involved risk-taking behaviour.

Participants were informed that if they were ranked among the top 10%, they would receive an additional two euros as a reward.

This incentive was designed to increase engagement and discourage participants from finishing the task hastily. Importantly, the competition was based on participants’ performance relative to other human participants (not the virtual agents), whose scores remained unknown during the task. Thus, the bonus was meant to promote individual effort rather than social comparison with the group.

### 2.5. Data analysis

As dependent measures, we investigated participants’ scores, the time they spent on each trial before quitting (hereafter referred to as quit time) and the number of times that their balloon burst (hereafter referred to as number of bursts). For each of these variables, we first considered only the factor of the number of involved agents with 40 trials at all four levels.

Then, to study the effect of agents’ risk-taking behaviour and its interaction with the number of involved agents on the participants’ risk-taking behaviour, we left out those trials in which none of the agents inflated their balloons and analysed the 120 remaining trials.

Furthermore, we investigated the trial-by-trial evolution of participants’ decision-making and its interaction with the social context by studying the participants’ scores through the experiment.

## 3. Results

### 3.1. Total score

#### 3.1.1. Analyses including zero agents

The Shapiro–Wilk test indicated significant deviations from normality for all agent conditions (*p* < 0.001), prompting the use of a non-parametric Friedman test. The test revealed a significant effect of the number of involved agents on participants’ scores, *χ*²(3) = 254.703, *p* < 0.001, Kendall’s *W* = 0.876, indicating a strong effect (*n* = 97). Mean ranks showed a consistent increase with group size (zero = 1.00, one = 2.07, three = 3.26 and five = 3.67), suggesting that participants scored higher when more agents were present.

**Figure 3.**
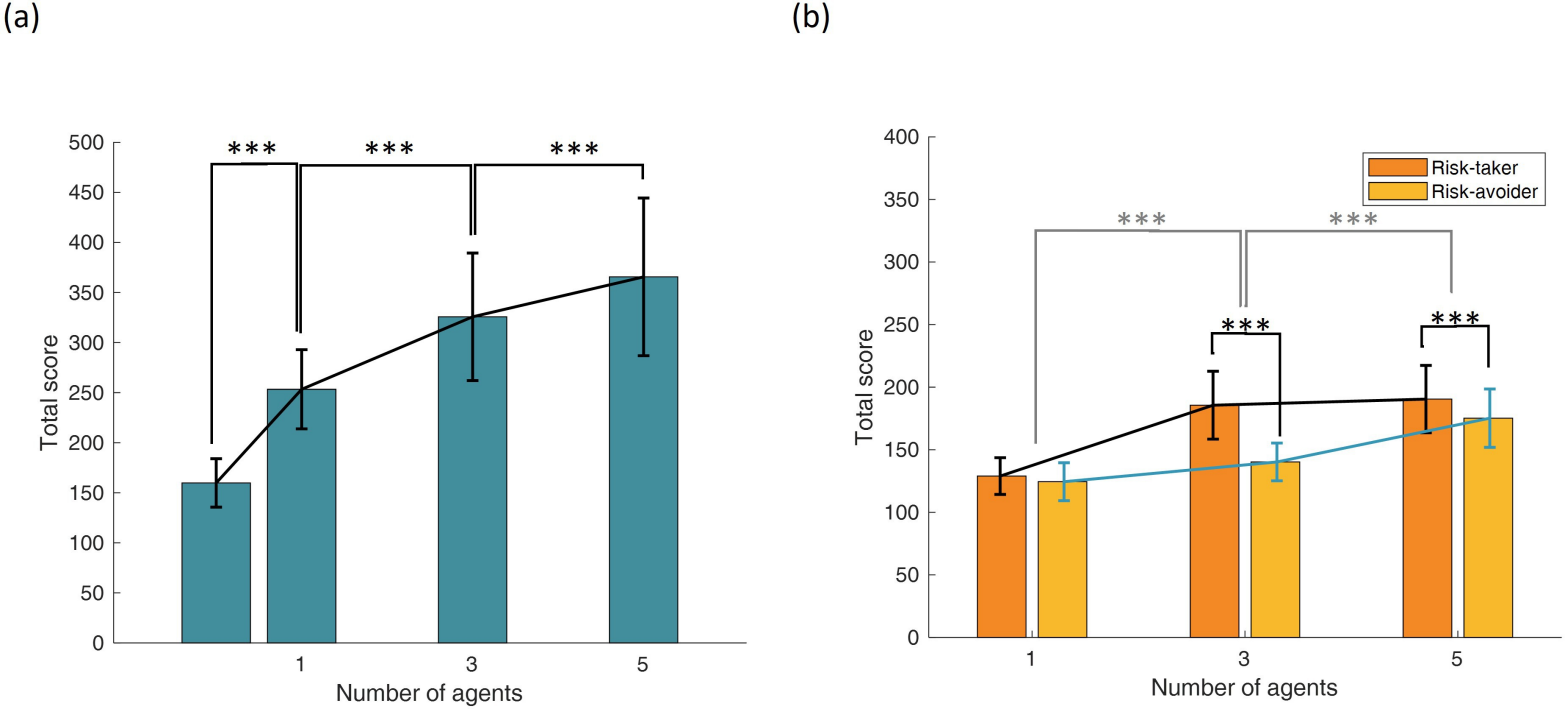
Total score (a) for zero, one, three and five involved agents and (b) for one, three and five agents in risk-taking and risk-avoiding conditions (error bars represent standard errors of the mean).

Pairwise, Wilcoxon signed-rank tests confirmed significant differences between all agent-number conditions (all *Z* < −4, all *p* < 0.001). These findings are visually summarized in figure 3a.

#### 3.1.2. Analyses without zero agents

To isolate the effect of the agents’ behaviour, we excluded trials in which no agents were inflating balloons and analysed the remaining 120 trials. Again, the data were non-normal (Shapiro–Wilk *p* < 0.001 for all conditions).

A Friedman test revealed a significant main effect of the number of involved agents on total score, *χ*²(2) = 133.505, *p* < 0.001, Kendall’s *W* = 0.688, suggesting a large effect. A Wilcoxon signed-rank test comparing total scores between risk-taking and risk-avoiding agent conditions was not significant, *Z* = −1.313, *p* = 0.189, *r* = 0.133.

To assess interaction effects, a Friedman test was conducted on the difference in scores between risk-taking and risk-avoiding conditions across agent numbers. This test yielded a significant result, *χ*²(2) = 46.479, *p* < 0.001, *W* = 0.239, indicating a moderate interaction effect.

Based on *post hoc* Wilcoxon signed-rank tests, there was no significant difference between risk-taking and risk-avoiding conditions when only one agent was involved. However, a significant difference was found in the three-agent condition (*Z* = −5.295, *p* < 0.001, *r* = 0.537) and in the five-agent condition (*Z* = −3.798, *p* < 0.001, *r* = 0.386). Furthermore, the risk-taking effect was significantly greater in the three-agent condition compared with the one-agent condition (*Z* = −4.036, *p* < 0.001, *r* = 0.410) and significantly smaller in the five-agent condition compared with the three-agent condition (*Z* = −6.836, *p* < 0.001, *r* = 0.694). These results are visualized in figure 3b.

### 3.2. Quit time

#### 3.2.1. Analyses including zero agents

**Figure 4.**
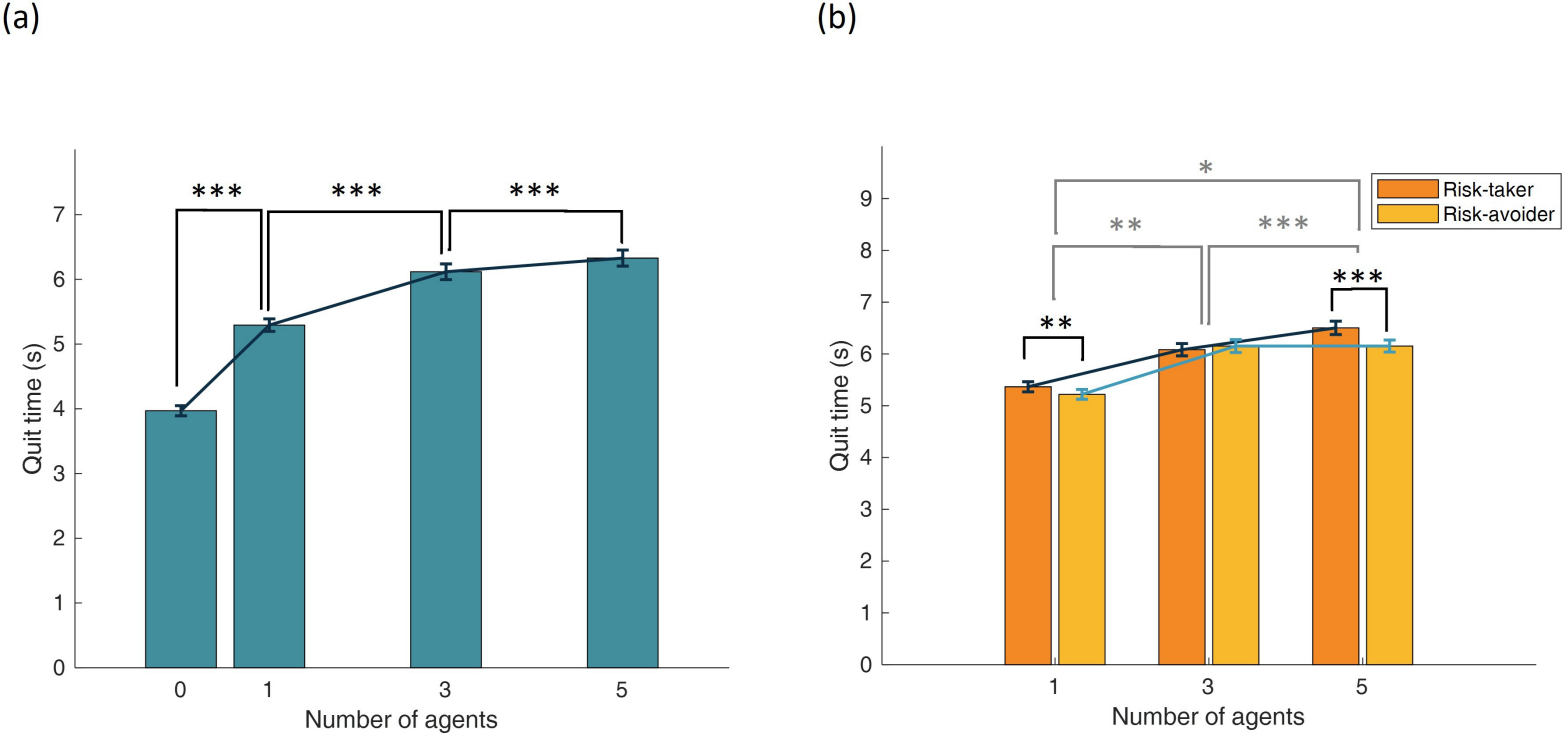
Quit time (a) for zero, one, three and five involved agents and (b) for one, three and five agents in risk-taking and risk-avoiding conditions (error bars represent standard errors of the mean).

Due to non-normal distributions (Shapiro–Wilk *p* < 0.001 for all), we conducted a Friedman test to evaluate the effect of agent number on participants’ quit times. Results showed a significant effect, *χ*²(3) = 253.429, *p* < 0.001, *W* = 0.871, indicating a strong impact of agent number on decision timing. Participants quit later with more agents present. Pairwise, Wilcoxon signed-rank tests showed significant differences between all conditions (all *Z* > 4.5, all *p* < 0.001). See figure 4a.

#### 3.2.2. Analyses without zero agents

A Friedman test again revealed a significant main effect of the number of involved agents, *χ*²(2) = 133.505, *p* < 0.001, *W* = 0.688.

A Wilcoxon signed-rank test comparing quit times between risk-taking and risk-avoiding agents showed a significant effect, *Z* = −3.883, *p* < 0.001, *r* = 0.394—participants quit significantly later in the risk-taking condition.

For interaction effects, a Friedman test was conducted on the difference in quit times between risk-taking and risk-avoiding conditions across agent numbers. The test yielded a significant result, *χ*²(2) = 10.917, *p* = 0.004, *W* = 0.056. *Post hoc* analyses showed that the difference in quit times was significantly greater in the five-agent condition compared with the one-agent condition (*Z* = −2.148, *p* = 0.032, *r* = 0.218). No significant difference was found in the three-agent condition. However, the difference between one and three agents was significant (*Z* = −1.97, *p* = 0.048, *r* = 0.200), and the difference between five and three agents was also significant (*Z* = −3.684, *p* < 0.001, *r* = 0.374). These results are shown in figure 4b.

### 3.3. Number of bursts

#### 3.3.1. Analyses including zero agents

Normality was confirmed. Mauchly’s test indicated a violation of sphericity, *χ*²(5) = 13.415, *p* = 0.020; thus, Greenhouse–Geisser correction (*ε* = 0.920) was applied.

**Figure 5.**
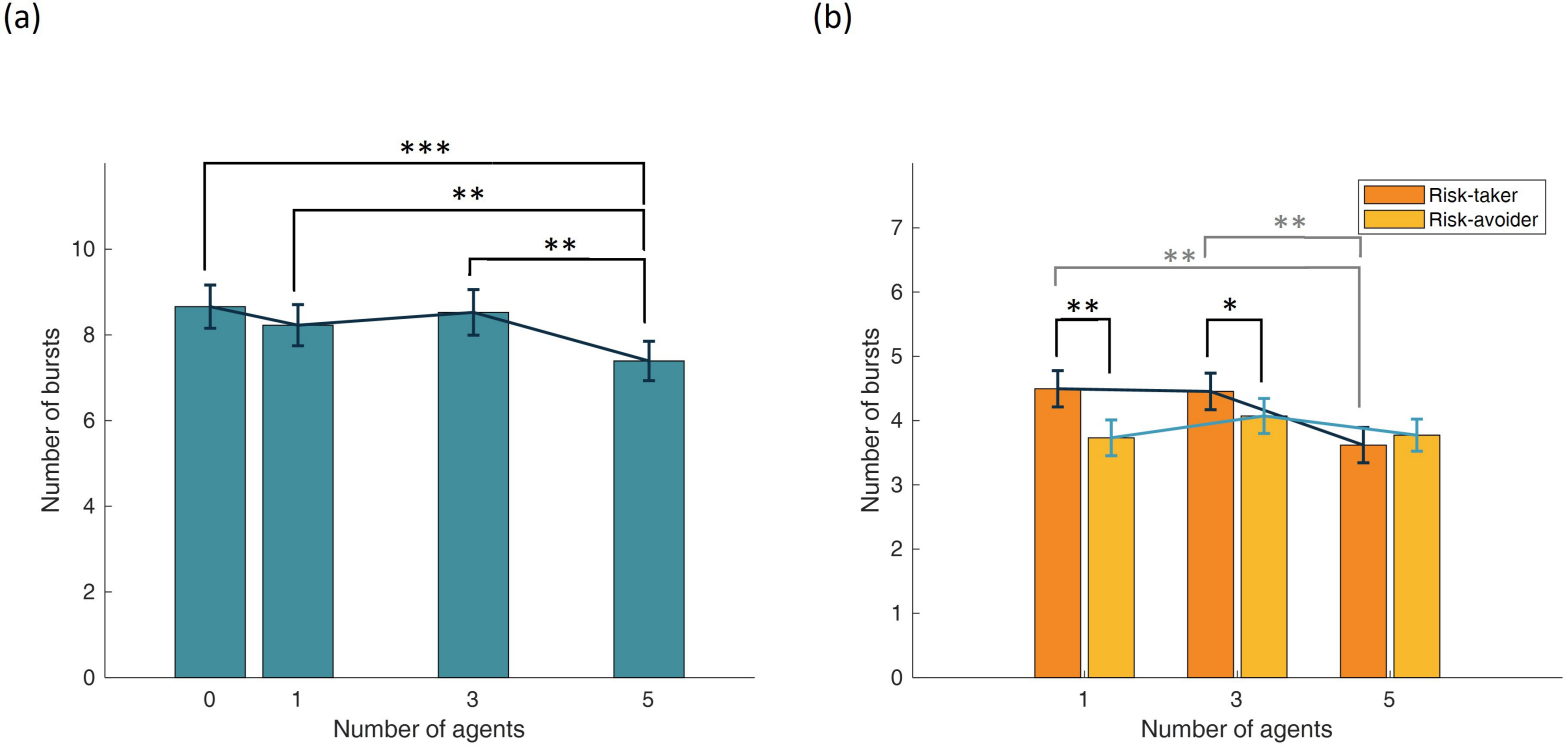
Number of bursts (a) for zero, one, three and five involved agents and (b) for one, three and five agents in risk-taking and risk-avoiding conditions (error bars represent standard errors of the mean).

A repeated-measures ANOVA revealed a significant main effect of agent number on the number of balloon bursts, *F*_3,288_ = 5.571, *p* < 0.001, *η*² = 0.055, such that participants experienced fewer bursts when more agents were involved. *Post hoc* Bonferroni-corrected comparisons showed that the number of balloon bursts was significantly lower in the five-agent condition compared with the zero-agent (*p* < 0.001), one-agent (*p* = 0.023) and three-agent conditions (*p* = 0.011), indicating a consistent decrease in bursts with increasing group size (figure 5a).

#### 3.3.2. Analyses without zero agents

A two-way repeated-measures ANOVA was conducted to examine the effects of agents’ risk-taking behaviour and its interaction with the number of involved agents on the number of balloon bursts. Due to violations of sphericity, Greenhouse–Geisser corrections were applied (*ε* = 0.902 for the agent number factor and *ε* = 0.940 for risk behaviour). The analysis revealed a significant main effect of agent risk-taking behaviour, *F*_1,96_ = 9.714, *p* = 0.003, *η*² = 0.092, indicating that participants experienced more balloon bursts in the risk-taking condition compared with the risk-avoiding condition. Additionally, a marginally significant interaction effect between agent number and risk-taking behaviour was observed, *F*_2,192_ = 2.993, *p* = 0.050, *η*² = 0.030.

*Post hoc* Bonferroni-corrected comparisons showed that participants experienced significantly more bursts in the risk-taking condition compared with the risk-avoiding condition when one agent (*p* = 0.010) or three agents (*p* = 0.022) were involved. Furthermore, the magnitude of this effect varied significantly across agent-number conditions, as confirmed by a separate analysis, *F*_2,95_ = 4.458, *p* = 0.010. These findings are illustrated in figure 5b.

### 3.4. Summary of findings

Taken together, these results demonstrate that participants’ risk-taking behaviour was strongly influenced by both the number and risk-taking behaviour of virtual agents. A larger number of involved agents—especially when agents demonstrated risk-taking behaviour—were associated with higher scores, longer quit times and fewer balloon bursts. The effect was most pronounced when three agents were involved, suggesting a nonlinear relationship between the number of involved agents and its influence.

## 4. Discussion

This study aimed to investigate how the risk-taking behaviour of a group influences individual risk-taking decisions in a dynamic task. The results indicate that participants’ risk-taking behaviour increased with the number of involved virtual agents. Furthermore, we consistently observed that higher risk-taking of the virtual group resulted in increased risk-taking by participants. Finally, we found a complex interaction between these two factors, revealing nuanced patterns in individual decision-making.

Our findings reveal that the number of involved agents significantly impacts individual risk-taking behaviour. As the number of involved agents increased, participants tended to take more risks, reflected in their higher scores and longer quit times. This result aligns with the concept of social influence and conformity, suggesting that acting together with others who are engaging in a similar task encourages individuals to push their boundaries, perhaps due to reduced perceived risk when acting together [42,43]. Interestingly, the decrease in the number of balloon bursts with an increasing number of involved agents reveals that even though participants showed riskier decisions when acting together with others, these decisions were nevertheless more optimal, leading to higher scores and fewer bursts than in a situation when participants act in a smaller group. This phenomenon might be partly due to the fact that participants could gain more information about the burst distribution when acting in a larger group and could thus reduce the number of bursts even when they were waiting longer before they quit and gained more points.

The phenomenon that individuals exhibit riskier behaviour in group settings compared with when alone has been well documented in previous literature [44,50,85]. However, our study provides a unique perspective by examining how individuals behave when surrounded by others in a group but make decisions independently, without direct communication or information exchange. This distinction makes our experiment particularly relevant for real-world scenarios of online decision-making, such as crossing a street against a red light. Research has shown that people are more likely to take risks in group situations [57,58,61,86,87], supporting the idea that acting together with others can embolden individuals even without explicit interaction or information sharing being minimal.

Moreover, we observed a nuanced relationship between the agents’ risk-taking behaviour and participants’ decisions. Participants performed riskier in trials where the virtual group showed riskier behaviour than in trials where the group was risk-avoiding. This observation echoes studies showing that individuals are more willing to engage in risky behaviour when peers are present, particularly when those peers model the behaviour [21,44]. However, we found a nonlinear pattern when comparing trials with different numbers of agents. This pattern strongly depended on the specific measure. For the total score, the difference between risk-taking and risk-avoiding conditions increased from one agent to three agents but decreased when five agents were involved, indicating that in a small group, the risk-taking of the group had the strongest influence. This seems to be due to the fact that with five risk-taking agents, the effect on the total score levelled out but still increased with five non-risk-taking agents. Previous research on social contagion already demonstrated that group influence shows an asymptotic trend, levelling out when the group becomes larger [88]. Interestingly, quit times and number of bursts showed a different pattern. However, these patterns were more difficult to interpret because both dependent measures strongly depend on each other.

The dynamic nature of our experiment is a crucial element that sets it apart from more static decision-making tasks. In this respect, our findings extend the literature by incorporating dynamic, fast-paced decision-making contexts, resembling real-life scenarios like driving, trading stocks or street crossing, where individuals must make split-second decisions based on continuously unfolding information. Similar to the real-world risky decision-making described in studies of jaywalking [57,58,61], participants in our study adjusted their behaviour based on the observed risk-taking behaviour of virtual agents.

These results contribute to the broader understanding of how social presence and group dynamics shape individual decision-making. As shown in prior work [50,89], acting in a group can increase individuals’ confidence and embolden riskier decisions, even when those others only provide minimal information or share responsibility for outcomes. Our study highlights this effect in a dynamic, observational context, extending these findings to scenarios where individuals operate independently of the group while still being influenced by its behaviour.

Furthermore, we found a marginally significant effect of trial progression, suggesting that participants’ behaviour slightly changed over time. More specifically, there was a weak trend towards increased risk-taking as participants advanced through the trials. This pattern may reflect a form of experiential learning in which individuals gradually adjust their strategy based on accumulating feedback. Even though this effect did not reach conventional levels of statistical significance, it indicates that participants may have become more confident or comfortable with the task structure, possibly calibrating their decisions based on perceived reward–risk tradeoffs over time. This aligns with previous findings from experience-based decision-making tasks, where learning from outcomes across repeated trials can influence future behaviour [90–92].

The implications of these findings extend beyond the laboratory. In real-world situations where people make individual decisions in the presence of others—such as investors on a trading floor or emergency responders during a crisis—the risk-taking behaviour of peers can heavily influence their decisions, even if they are not working collaboratively. This aligns with research on social influence and responsibility diffusion, which suggests that individuals may take greater risks when the perceived burden of adverse outcomes is distributed among a group [34,36]. Understanding these dynamics can help inform strategies for managing group risk in high-stakes environments, such as improving safety protocols or designing interventions to reduce excessive risk-taking in certain settings.

An important methodological contribution of our study lies in its use of an online, multi-agent simulation to examine social influence in dynamic risk-taking tasks. Unlike traditional laboratory-based paradigms—which require the simultaneous presence of multiple participants and careful synchronization—our approach allowed for a high degree of control and scalability while preserving the illusion of real-time group interaction. While VR or desktop-based multi-player BART implementations could offer greater ecological realism or social presence, they often come with substantial logistical and technical demands. In contrast, our design balances ecological validity and experimental control, enabling the study of group effects without the need for physical co-presence or complex hardware. Moreover, it opens up possibilities for collecting data from large and diverse samples across locations, making it particularly useful for future research in distributed environments such as online education, remote teamwork and digital collaboration platforms.

While our setup may not replicate the exact physical conditions of real-world group decision-making scenarios (i.e. limited mundane realism), it was designed to maximize experimental realism—ensuring that participants felt psychologically engaged and perceived the agents as socially relevant actors. Prior research suggests that experimental realism is more critical than physical realism in evoking authentic behavioural responses in social and risk-related tasks [93]. Our use of realistic avatars and naturalistic motion cues served this goal by creating a credible and immersive social context.

In conclusion, our study provides important insights into how both active group size—the number of people engaged in the same task—and their risk-taking behaviour influence individual risk-taking tendencies in dynamic environments. These findings support the idea that social influence plays a critical role in shaping risk-related decisions, even when individuals are not directly interacting with others. Future research could further explore how varying levels of information-sharing or group visibility affect risk-taking behaviour, and how individual differences, such as susceptibility to peer influence, may moderate these effects. Additionally, it would be valuable to examine how these dynamics play out in more interactive scenarios implemented in multi-player settings where participants can observe each other’s behaviour in real time, offering even closer parallels to real-world risky decision-making environments.

## Data Availability

The data supporting the findings of this study are openly available in the Open Science Framework [94].
